# Impact of Phenolic Compounds on Fibrillogenesis of Human Lysozyme under Physiological Conditions: Inhibition or Promotion?

**DOI:** 10.1021/acsomega.5c02787

**Published:** 2025-08-05

**Authors:** Santos López, Arturo Rojo-Domínguez, Hugo Nájera

**Affiliations:** † Posgrado en Ciencias Naturales e Ingeniería, 27786 Universidad Autónoma Metropolitana—Cuajimalpa, Av. Vasco de Quiroga 4871, Colonia Santa Fe Cuajimalpa, Alcaldía Cuajimalpa de Morelos, Ciudad de México 05348, Mexico; ‡ Departamento de Ciencias Naturales, Universidad Autónoma Metropolitana—Cuajimalpa, Av. Vasco de Quiroga 4871, Colonia Santa Fe Cuajimalpa, Alcaldía Cuajimalpa de Morelos, Ciudad de México 05348, Mexico

## Abstract

Numerous compounds have been studied in the search for a potential drug to interrupt the process of formation of the amyloid fibers. Human lysozyme is a good model for studying the formation of amyloid fibers. In this research, the effect of phenolic compounds (caffeic acid, l-tyrosine, pyrogallol, guaiacol, 6-(*p*-toluidino)-2-naphthalenesulfonic, epicatechin, chrysin, quercetin) during the formation of amyloid fibers at physiological conditions (37 °C and pH 7.5) was studied, resulting in inhibition with certain compounds; on the contrary, the formation of fibers was favored by others. Fluorescence experiments were carried out, like thioflavin T and 8-anilino-1-naphthalenesulfonic acid, where the signal change indicates an increase or a decrease of the amyloid fibers. Circular dichroism was made to understand the changes in the second structure produced for the interaction of the phenolic compounds with the lysozyme. Additionally, molecular docking experiments indicate that the interaction of the compounds with specific amino acids of lysozyme is crucial for inhibiting or exerting a higher effect on fiber formation.

## Graphical abstract

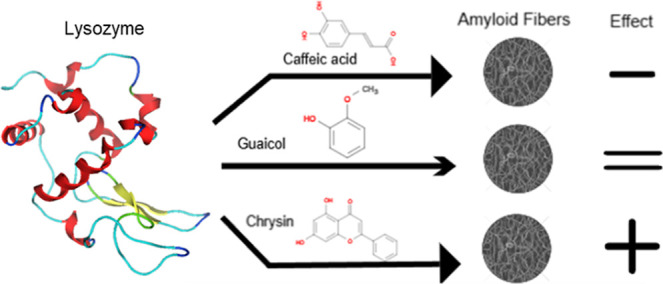

## 1 Introduction

Human lysozyme is a widely studied protein; for this reason, it is one of the most used models for studying the folding process. The first structure report was in 1965; the most recent is from 2022.^1^ This protein is considered fibrillar because it causes a disease known as hereditary lysozyme amyloidosis; three mutations are related: Asp67His, Ile56Thr,^2^ and the last Phe57Ile.^3^ Some characteristics are a molecular weight of 16.5 kDa, two domains, one α and the other β. The tertiary structure is compact and globular with a long indentation on its surface. Some tryptophans are in the binding site; these are Trp-64 and Trp-109. They have two functions: the first is binding the substrate, and the second is establishing the structure of the protein.^4^

Proteins are essential molecules for life; they perform numerous functions. For this reason, having good control of their quality is important. For the correct function of a protein, it is necessary to have a correct environment and good folding.^5^ When proteins undergo an incorrect folding process, it is possible to obtain an abnormal protein assembly known as amyloid fibers.^6^ These fibers consist of self-assembled aggregates of fibrous protein and are linked to various currently incurable conditions, such as Alzheimer’s and Parkinson’s diseases that affect the central nervous system. Millions of individuals around the globe are affected by amyloid diseases.^7^ Additionally, there are systemic forms of amyloidosis in which the protein forms insoluble deposits in various organs and tissues. Over 40 different proteins have been identified as amyloid fibers.^8^ The structure of these amyloid fibers is mainly characterized by a cross-β conformation, which provides a high level of stability against degradation. Another notable feature is their binding with Congo red dye; when this occurs, the fiber exhibits a birefringence that appears to be an apple-green color and can be observed under a polarized light microscope.^9^

The most common method for studying the formation of amyloid fibers is the use of thioflavin T (ThT).^10^ ThT is a cationic benzothiazole; its most important characteristic is the molecule rotation. The mechanism for detecting the amyloid fibers with ThT consists of the binding of the fluorophore to the channels formed for the β-sheets. This binding causes ThT to become rigid there before the fluorescence appears.^11^

Research aimed at identifying compounds capable of inhibiting amyloid fiber formation has tested numerous molecules, with phenolic compounds emerging as a particularly promising group.^12^ These compounds have been demonstrated to have great potential to inhibit fibrillogenesis;^13^ however, on the other hand, some promote the formation of amyloid fibers, as shown in this work.

## 2 Materials and Methods

### 2.1 Compounds

Thioflavin T, human lysozyme, l-tyrosine (l-tyr), caffeic acid, pyrogallol, guaiacol, 6-(*p*-toluidino)-2-naphthalenesulfonic acid (6-pT), epicatechin, chrysin, quercetin, and 8-anilinonaphthalene-1-sulfonic acid were sourced from Sigma-Aldrich.

### 2.2 Amyloid Fiber Formation

Human lysozyme was prepared in a stock solution at a concentration of 50 mg mL^–1^ in 20 mM potassium phosphate buffer, pH 7.44, which was used for all experiments. ThT was prepared as a 10 mM stock solution in absolute ethanol. Fibrillogenesis was assessed using a Costar 96-well plate and monitored by ThT fluorescence in a plate reader (TECAN Infinite M1000Pro). The final concentrations in each well were 1725 μM lysozyme and 66 μM ThT, all in the potassium phosphate buffer. Samples were excited at 450 nm, with emission detected at 490 nm. Kinetics were measured every 10 min for 6 h at 37 °C.

### 2.3 Fibrillogenesis in the Presence of Phenolic Compounds

All phenolic compounds were prepared as a 2.5 mM stock solution. Inhibition assays were conducted using a 1:1 molar ratio of lysozyme to phenolic compound. Fibrillogenesis was monitored using ThT fluorescence in a plate reader (TECAN Infinite M1000Pro) under the same conditions as described. The final concentration was 66 μM of ThT, all in the potassium phosphate buffer. Excitation was at 450 nm, with emission measured at 490 nm. Kinetics were recorded every 10 min for 6 h at 37 °C.

### 2.4 ANS Assay

ANS was prepared as a 5 mM stock solution in potassium phosphate buffer. Two experimental conditions were evaluated: (1) amyloid fiber formation with lysozyme alone at a final concentration of 1725 μM, and (2) in the presence of phenolic compounds at a 1:1 molar ratio with lysozyme. ANS was used at a final concentration of 60 μM for both conditions. ANS fluorescence was monitored using a plate reader (TECAN Infinite M1000Pro). Samples were excited at 365 nm, with emission detected at 450 nm. Kinetics were recorded every 10 min for 8 h at 37 °C.

### 2.5 Circular Dichroism (CD)

CD spectra were recorded for native human lysozyme, amyloid fibers of human lysozyme, and the result of the fibrillogenesis in the presence of the phenolic compounds using a Jasco J-815 CD spectropolarimeter (Tokyo, Japan) over the range of 260 to 190. All CD spectra reported are the average of three consecutive scans. CD spectra data were analyzed using the “BESTSEL” program.

### 2.6 Scanning Electron Microscopy (SEM)

The microscopy was conducted with a Hitachi TM3030Plus microscope. A drop was deposited on a glass coverslip to prepare the samples and permitted to dry for 24 h. After drying, the sample coverslip was secured onto the microscope stage using carbon tape. All samples were examined following 16 h of incubation.

### 2.7 Graphics and Data Processing

All graphics and data processing were made with Origin 2016.

### 2.8 Docking

Computational docking studies were conducted using molecular operating environment (MOE version 2007). The structure of human lysozyme was obtained from the Protein Data Bank (PDB) (ID 3FE0). All water was removed for structure preparation, hydrogen atoms were energy minimized, and partial charges were assigned according to the CHARMM27 force field. The ligands were obtained from PubChem, and the MMFF94x force field was used to assign partial charges for each compound. The entire protein surface was used to define the interaction site and pose search with the Alpha triangle method. Up to 15,000 potential poses were assayed in each computational experiment. The scoring function employed was London dG, and the top 30 results were refined using this function to optimize the poses. Interaction maps were generated using MOE analysis tools to obtain the best result for each docking experiment.

## 3 Results and Discussion

### 3.1 Effect of Phenolic Compounds in Fibrillogenesis of Human Lysozyme

The effect of phenolic compounds on the fibrillogenesis of human lysozyme at physiological conditions was investigated. The study focused on how various phenolic compounds affect the formation of amyloid fibers under physiological pH and temperature conditions. The formation of amyloid fibrils was monitored using ThT fluorescence. Eight compounds were examined: caffeic acid, l-tyr, pyrogallol, guaiacol, 6-pT, epicatechin, chrysin, and quercetin. Three distinct effects were observed and are showed in Figure 1A. An increase in fluorescence indicated a more significant appearance of amyloid fibers, with epicatechin producing the most significant increase, suggesting it is a poor inhibitor of fibrillogenesis. This finding aligns with previous reports on islet amyloid polypeptide (IAPP, also known as amylin).^14^ Other compounds contributing to increased fluorescence included guaiacol, chrysin, and pyrogallol. Conversely, 6-pT and quercetin exhibited fluorescence levels similar to lysozyme, indicating no effect on fibrillogenesis. Lastly, a decrease in fluorescence was noted, signaling an inhibitory effect on fibrillogenesis,^15^ which has been documented for certain phenolic compounds in hen egg-white lysozyme under different conditions.^16,17^

**Figure 1:**
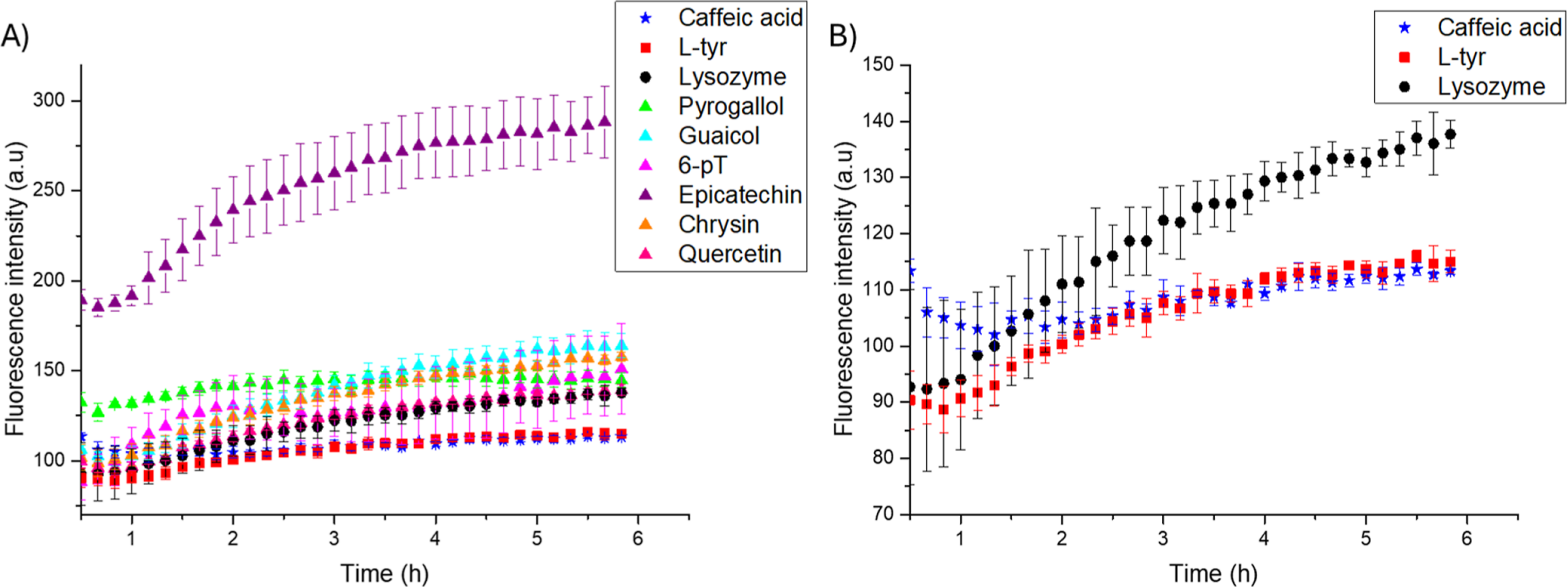
(A) Effect of phenolic compounds in the formation of amyloid fibers. Black (lysozyme), red (l-tyr), blue (caffeic acid), green (pyrogallol), cyan (guaiacol), magenta (6-pT), purple (epicatechin), orange (chrysin), and pink (quercitin). (B) Lysozyme and the compounds with inhibitory effect, black circles (lysozyme), red squares (l-tyr), and blue stars (caffeic acid). The molar ratio 1:1 (protein: compound) was tested in all cases.

Figure 1B compares lysozyme and two compounds exhibiting inhibitory effects, caffeic acid and l-tyr. The results demonstrate that their effects are similar, as evidenced by the comparable fluorescence observed after 2 h, which persists until the end of the experiment. Caffeic acid has been reported to both prevent and treat Alzheimer’s disease^18^ while derivatives of l-tyr have been identified as inhibitors of amyloid-beta aggregation.^19^

These findings suggest that both molecules are promising foundations for discovering therapeutic agents targeting various diseases.

Some studies indicate that the differing effects induced by phenolic compounds can likely be attributed to subtle structural differences among them, including variations in the phenolic rings, functional groups, charge, and size.^20,21^ Previous research has also highlighted the correlation between biological activity and the number of hydroxyl groups in these compounds.^22^ Hence, docking experiments were conducted to gain further insights.

### 3.2 8-Anilino-1-naphthalenesulfonic Acid (ANS) Assay

To determine whether the inhibitory effects of caffeic acid and l-tyr are comparable, hydrophobicity was assessed using ANS, which interacts with the hydrophobic regions of proteins or their aggregates.^23^

Figure 2A illustrates that the fluorescence intensity of ANS significantly decreases in the presence of inhibitory compounds. This observation is consistent with the outcomes of ThT assays, which indicate that the fluorescence intensity of the dye directly correlates with the presence of amyloid fibers.^24^

**Figure 2:**
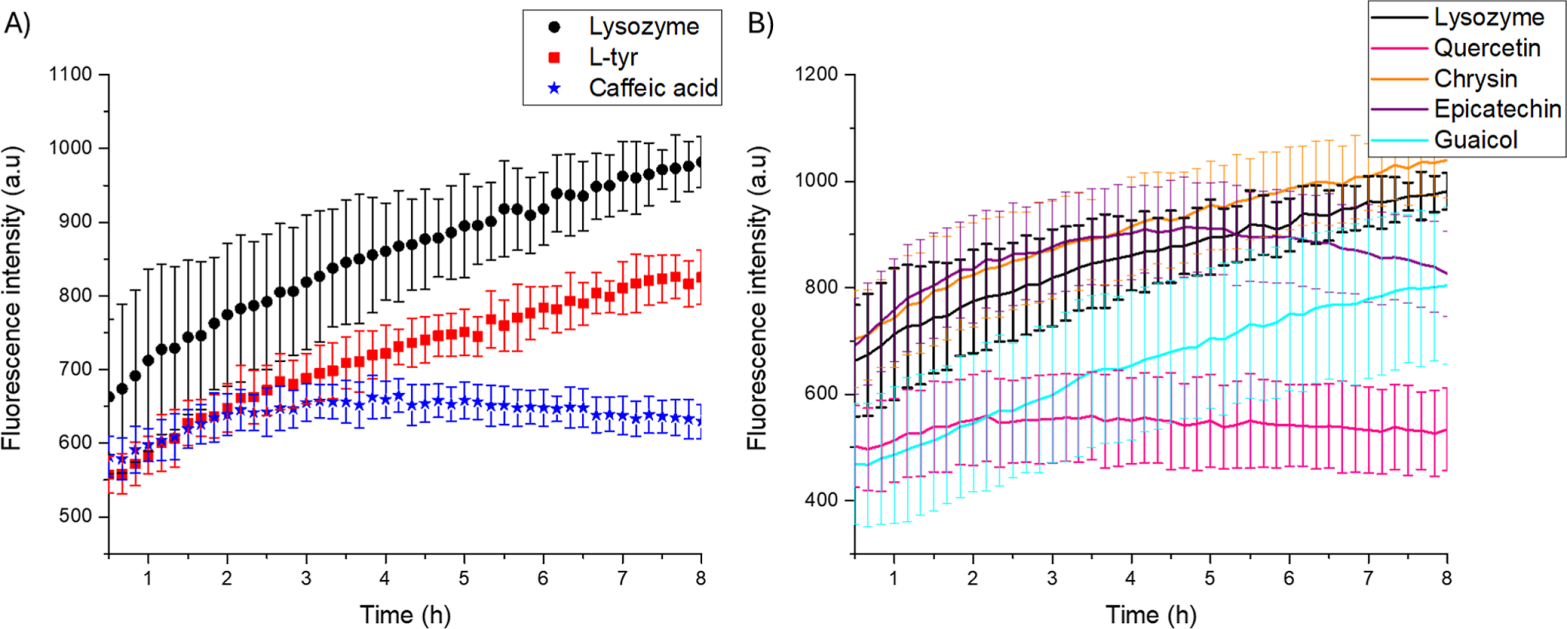
Effect of phenolic compounds in the amyloid fiber formation of lysozyme with ANS as a reporter. (A) Compounds with inhibitory effect l-tyr and caffeic acid. Lysozyme alone (black circles), with l-tyr (red squares), and with caffeic acid (blue stars). (B) Lysozyme in the presence of other compounds: quercetin (pink), chrysin (orange), epicatechin (purple), and guaiacol (cyan). Lysozyme in the presence of 6-pT and pyrogallol are absent because their fluorescence is over 1700 units.

The decrease in fluorescence indicates that fewer hydrophobic regions are exposed. However, when comparing the two compounds, caffeic acid demonstrates a more significant effect than l-tyrosine. This suggests that their inhibitory effects are not identical; although both start similarly, they begin to diverge after 2 h. These observations indicate an early interruption in the fibrillogenesis process for both compounds. However, they reveal differences in the later stages of this process despite both being classified as inhibitors. Additionally, the results show that lysozyme with l-tyrosine exposes more hydrophobic regions than lysozyme with caffeic acid, and both differ from the amyloid fibers formed from lysozyme alone.

On the other hand, Figure 2B displays the results for other phenolic compounds tested. In this panel, lysozyme with 6-pT and pyrogallol are absent because their fluorescence exceeds 1700 units. Notably, only quercetin (represented in pink) reduces exposure to hydrophobic areas. However, in the ThT experiment, its fluorescence matches that of lysozyme, suggesting a different mechanism for forming amyloid fibers and a distinct final structure for the fibers. To other compounds, their ANS fluorescence levels are similar to those of lysozyme, as indicated by the ThT experiments. This suggests that the processes for forming amyloid fibers may be consistent across these compounds.

### 3.3 Circular Dichroism

Circular dichroism is a spectroscopic method employed to examine the changes in secondary structure changes of proteins.^25^ When a native protein passes to an amyloid fiber, a change in structure occurs, losing its α-helix and change to a β-sheet conformation.^26^ Figure 3 shows the various structural changes in lysozyme during fibrillogenesis in the presence of different phenolic compounds. It can be observed that the final structure of the protein differs depending on which compound is present; however, neither structure resembles the native lysozyme form (gray) nor its fibrillar form (black). A negative peak distinguishes native human lysozyme at 208 nm and a negative shoulder at 222 nm.^27^

**Figure 3:**
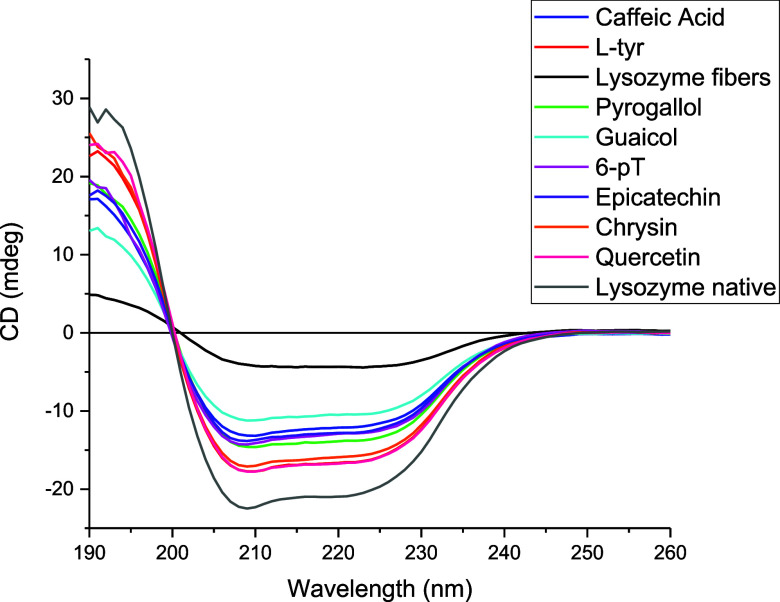
Far–UV CD spectral changes of lysozyme during fibrillation and the final structure after the effect of phenolic compounds in the fibrillogenesis.

After an 8 h incubation period, fibrillar lysozyme (black) shows a loss of the native spectrum. Table 1 shows the structural change from native lysozyme to amyloid fibers, where α-helix passes to 0%, β-sheet changes to 40.1%, the turns pass to 14.6%, and others pass to 45.3%. These changes show an increase of others, turns and β-sheet and the loss of α-helix that match with the expected when a protein becomes an amyloid fiber. This change in the structure is common at smaller scales and time scales and is the first to appear in many amyloid aggregation processes.^28^

**Table 1:** CD Analysis for Lysozyme, Lysozyme Fibers, and Lysozyme in the Presence of Different Phenolic Compounds

parameter/compound	lysozyme fibers	caffeic acid	l-tyr	6-pT	chrysin	epicatechin	guaiacol	pyrogallol	quercetin	native lysozyme
α helix	0	15.6	22.9	14.6	21	15.9	13.5	17.3	21.7	53.4
β-sheet antiparallel	40.1	21.3	15.4	22.2	17.9	21.5	24.3	20	18.4	11.8
β-sheet parallel	0	8.4	7.9	6.3	7.8	7.9	5.2	7.8	7.7	2.1
turn	14.6	12.8	13.3	14.2	13	13.5	13.5	13.9	13.2	3.8
others	45.3	41.9	40.6	42.8	40.4	41.1	43.5	41	39	28.8

When l-tyr is present, lysozyme conserves a significant part of the original distribution of the structure. The result suggests that the final structure of the lysozyme in the presence of l-tyr is different from the amyloid fibers and the native protein.^29^

Caffeic acid is the other compound with an inhibitory effect that shows different values when present in the fibrillogenesis than native lysozyme, fibers, and lysozyme in the presence of l-tyr. α-Helix parameter is in the middle of the values with 15.6%, the same as β-sheet structures, turns it is the unique value (12.8%) where it is the lowest of all the conditions, and for others, it is in the average. This means that the way to inhibit fibrillogenesis differs from the l-tyr. The result obtained for ThT suggests that the inhibitory power is the same for both compounds. Still, ANS and CD analysis show that the final structure of the inhibition is different from that earned with l-tyr.

The results for the other six compounds indicate that the α-helix parameter is lower than that of native Lysozyme but higher than that of lysozyme fibers. In terms of the total β-sheet content (which includes both antiparallel and parallel sheets), the values are as follows: 6-pT at 28.5%, chrysin at 25.7%, epicatechin at 29.4%, guaiacol at 29.5%, pyrogallol at 27.7%, and quercetin at 26.1%. These percentages are closer to the 40% found in amyloid fibers. A similar trend is observed with the turns.

These findings suggest that the presence of phenolic compounds does not completely transform the protein into amyloid fibers but instead leads to the formation of partially structured fibers. This conclusion is supported by the fluorescence observed in the ThT and ANS assays. The ANS assay shows that the hydrophobic zones are similar, whereas ThT shows a fluorescence change. This difference may be attributed to variations in the surface charge of the amyloid fibers, as a negative surface charge attracts the ThT dye, resulting in a stronger affinity to the fibrils and, consequently, higher fluorescent intensity.^30^

### 3.4 Scanning Electron Microscopy (SEM)

For microscopy, three compounds were selected, a representative of each case for the inhibitory caffeic acid was selected, for the promoter effect epicatechin, and for the apparently no effect 6-pT.

SEM results are present in Figure 4A, which shows the structure that results of the inhibitory effect, it is possible to see two small fibers in the center of the image. Figure 4B shows a 6-pT effect where a more significant amyloid fiber is present, and Figure 4C presents the promoter effect for the best compound; here, it shows a full amyloid fiber. These results suggest that the difference in the effect is directly related to the size of the fiber, and if CD results are taken into account, probably the structure is different and is the principal reason for these results. Lysozyme alone and l-tyr are different from those results published in our previous work.^29^

**Figure 4:**
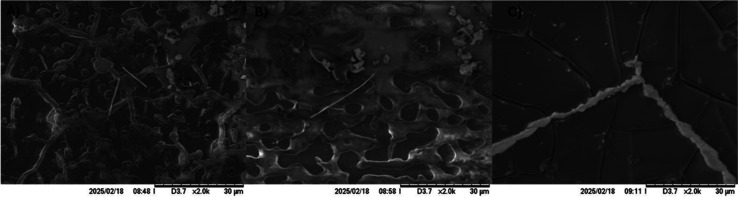
SEM images of lysozyme after the fibrillogenesis in the presence of different compounds. (A) Lysozyme in the presence of caffeic acid. (B) Lysozyme in the presence of 6-pT. (C) Lysozyme in the presence of epicatechin. All were obtained at 5 kV in BSE mode.

### 3.5 Docking

Bioinformatics provides valuable insights into the underlying interaction mechanisms, offering more detailed information. Docking experiments can identify the likely binding sites where a compound interacts with a protein.^31^ For this research, PBD ID 3FE0 was used as a model for human lysozyme, and all compounds were obtained from PubChem; both were prepared as described in the material methods section. All protein was used in the search of the interaction site, and the result shows two zones in the protein where the compounds are binding, as shown in Figure 5.

**Figure 5:**
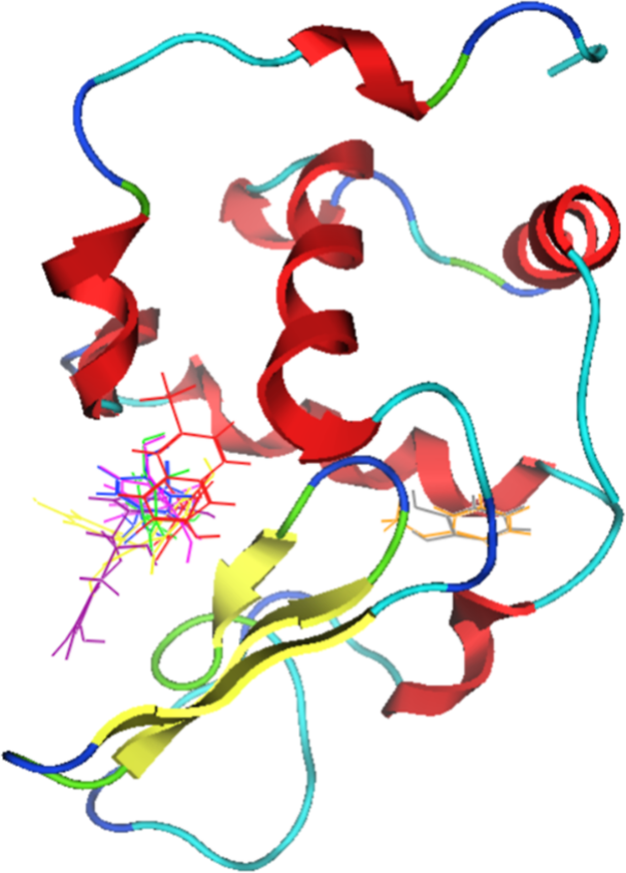
Human lysozyme docked with phenolic compounds, 6-pT (red), caffeic acid (green), chrysin (pink), epicatechin (purple), guaiacol (orange), l-tyr (blue), pyrogallol (gray) and quercetin (yellow).

At the active site, where 6-pT, caffeic acid, chrysin, epicatechin, l-tyr, and quercetin are attached, it is interesting to note that each compound has a unique orientation. This diversity in orientations suggests that the interactions with lysozyme could be distinct in each case. On the opposite side, guaiacol and pyrogallol exhibit a preference. Both zones could interact with the lysozyme alpha and beta domains. Importantly, this suggests that an interaction between both domains influences fibrillogenesis. This zone is lost when lysozyme converts to amyloid fibers, because the lysozyme loses its α domain and changes to a β-sheet structure, becoming planar.^32,33^ Hence, interacting in these areas can be crucial for inhibiting or promoting the formation of fibers.

l-Tyrosine and caffeic acid, known for their significant inhibitory effects, share almost all parameters, as shown in Figure 6. Their molecular weights are 181.19 g/mol and 189.16 g/mol, respectively. Both compounds contain a single ring and have short carbon chains. The docking energies are −13.38 kcal/mol for l-tyrosine and −12.51 kcal/mol for caffeic acid.

**Figure 6:**
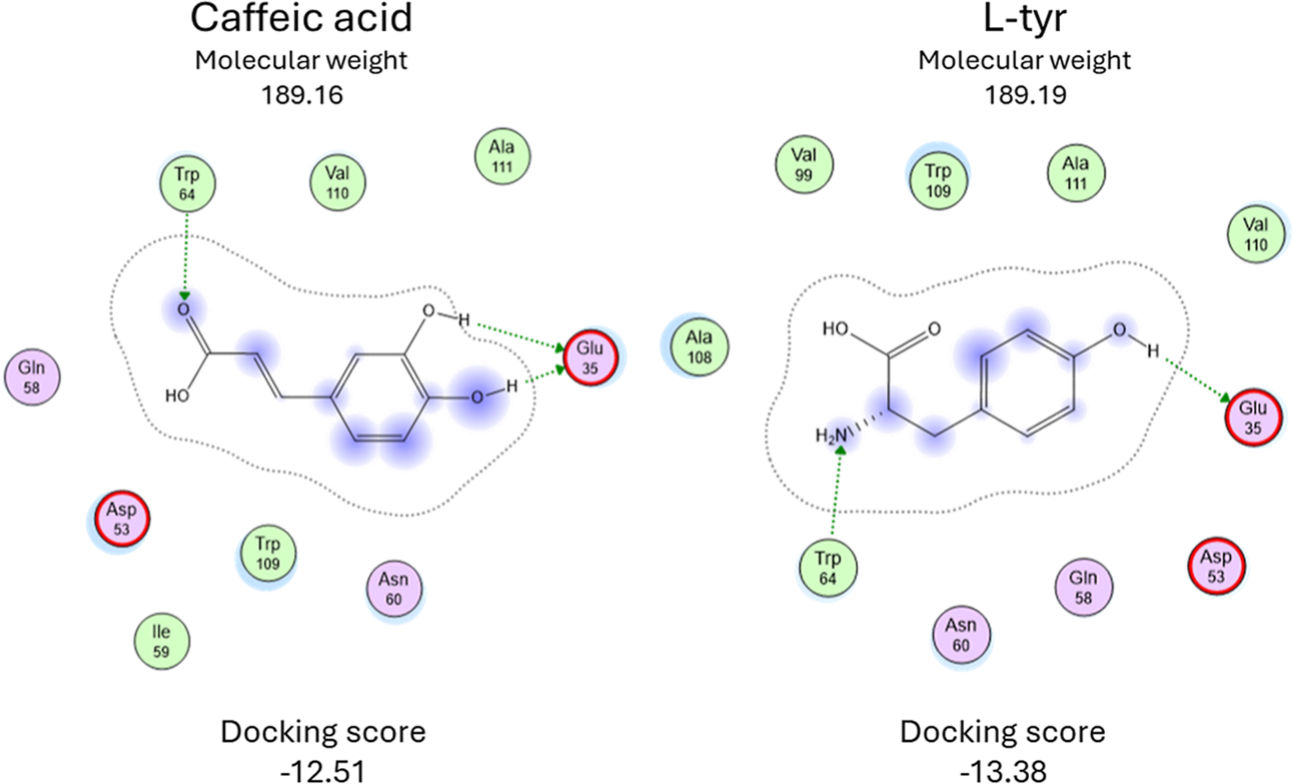
Interaction map of the best docking results for caffeic acid and l-tyr.

In terms of interactions, both compounds engage with identical residues: Glu35 forms hydrogen bonds with the hydroxyl groups on their rings. Additionally, l-tyrosine interacts with the amino group of Trp64, while caffeic acid interacts with the carboxyl group of the same residue. These similarities suggest that these interactions with the two specific amino acids are sufficient to produce an inhibitory effect. Notably, Glu35 is one of the critical residues for enzymatic activity,^34^ indicating its significant structural role. Additionally, these amino acids were predicted with a high aggregation tendency,^32^ so stabilizing them could be the key for the inhibit effect.

Guaiacol and pyrogallol are other compounds of interest because both prefer a different zone than the other phenolic compounds. These two molecules are the smallest; their MW is 124.14 and 126.11, respectively. They have only one phenolic ring without R chains, and their energy is similar at −9.06 and −10.64, but the interactions are slightly different. Figure 7 shows that guaiacol forms a single hydrogen bond with Ala83, and Pyrogallol makes two, the first with Ile56 and the second with Ala83. Interaction in the other zone is because these two small molecules can enter a smaller cavity. Both molecules produce a similar effect compared to the more prominent compounds. This suggests that interaction with Ala83 could be a key amino acid for promoting the formation of amyloid fibers.

**Figure 7:**
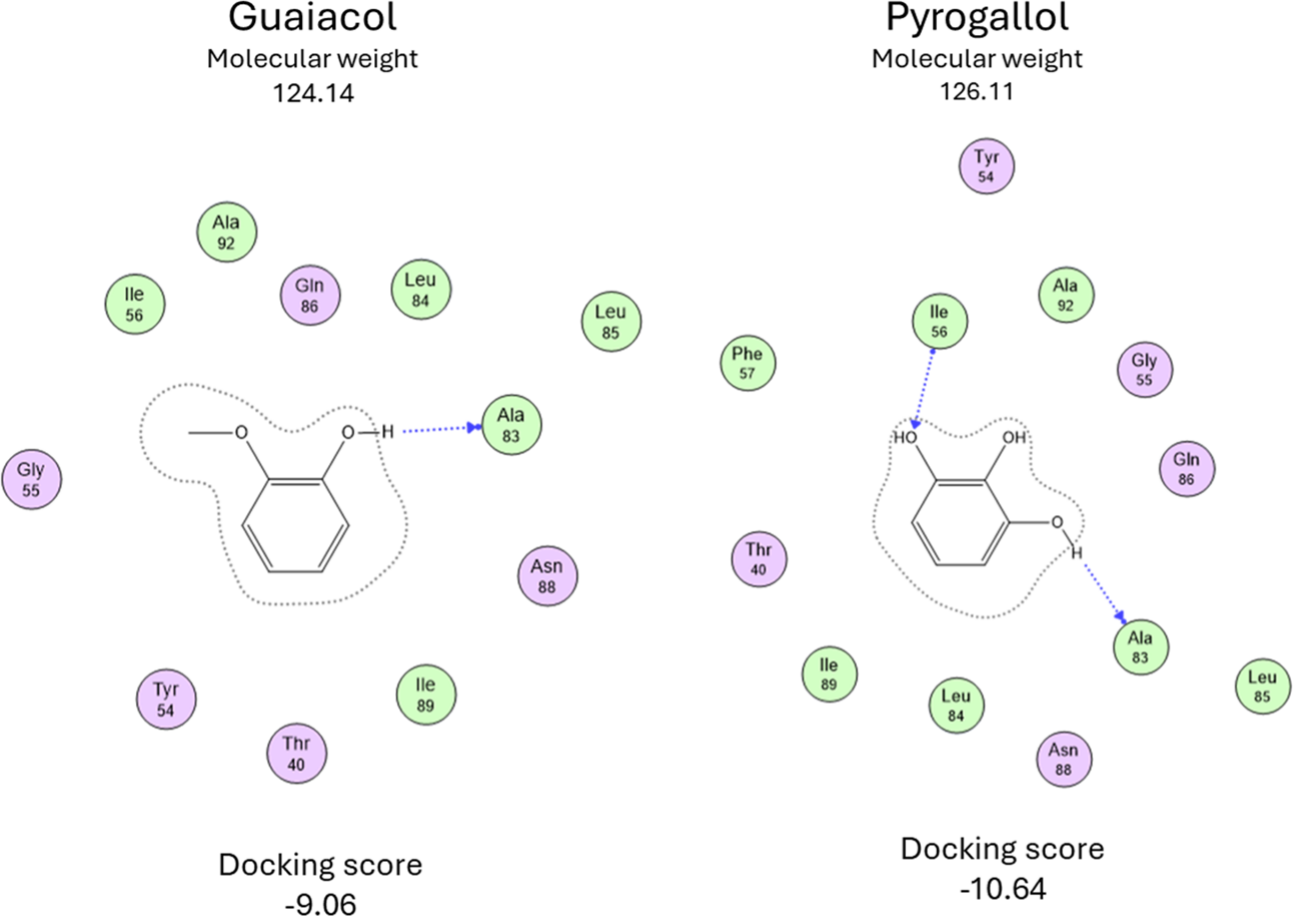
Interaction map of the best docking results for guaiacol and pyrogallol.

Regarding the other four compounds shown in Figure 8 (6-pT, chrysin, epicatechin, and quercetin), it was found that they share only two parameters; specifically, their molecular weight (MW) they are the heaviest of the analyzed compounds over 290.27 g/mol and their number of phenolic rings all have three. Energy and interactions differ between them.

**Figure 8:**
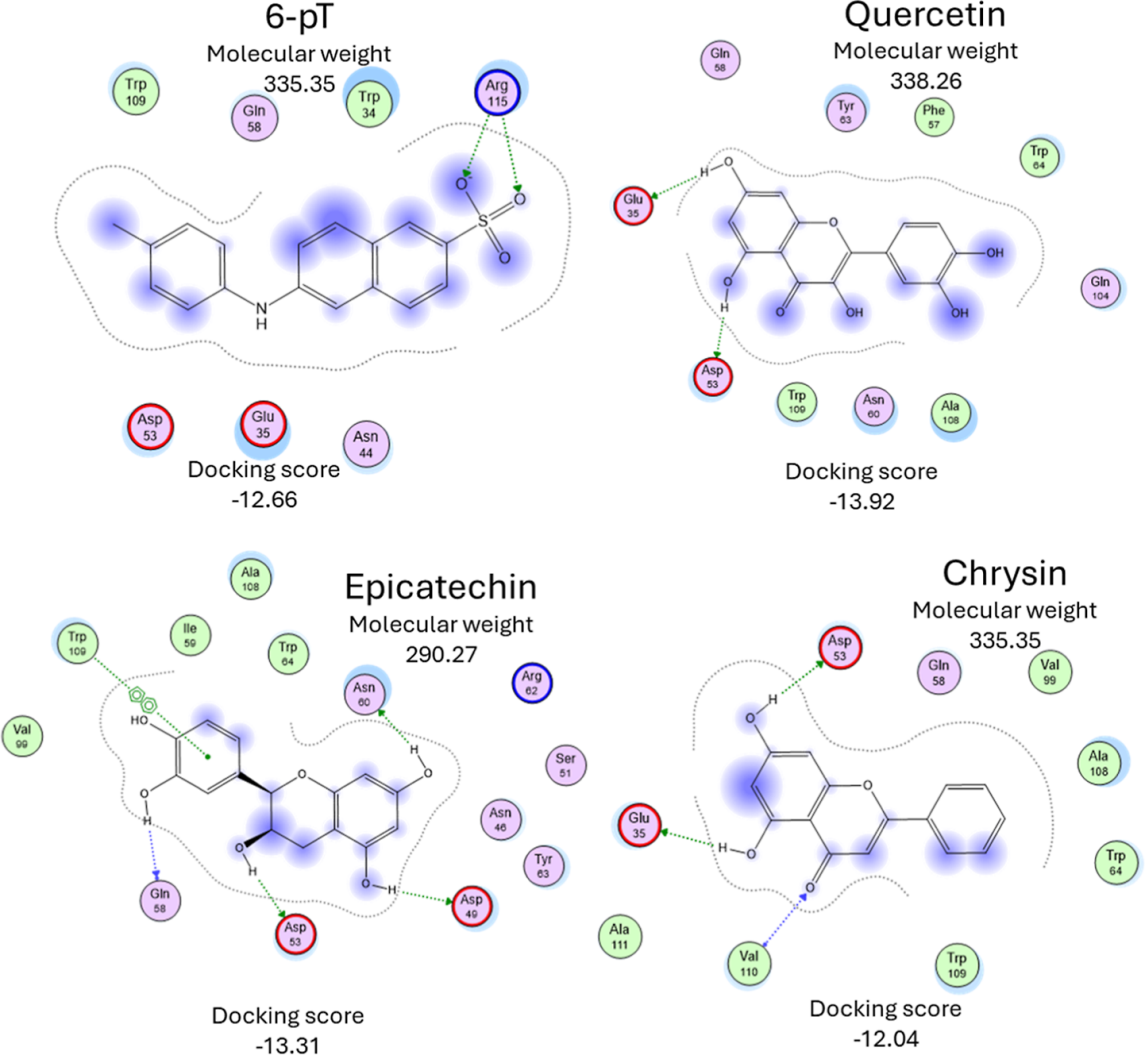
Interaction map of the best docking results for 6-pT, chrysin, epicatechin, and quercetin.

The difference in structure makes them too large to interact with both Glu35 and Trp64 simultaneously. In contrast, the interactions are different in any case; for 6-pT, two hydrogen bonds are present with Arg115; this interaction does not have an effect because Tht, ANS, and SEM experiments suggest that it forms a normal amyloid fiber. Quercetin has two interactions, first with Glu35 and Asp53; chrysin is similar but has an additional hydrogen bond with Val110; this result indicates that only interaction with Glu35 is not enough to have an inhibitory effect. This suggests that both interactions must be present to prevent fibrillogenesis.

For epicatechin, Figure 8 shows that it is the compound with the most interactions and has four hydrogen bonds, distributed in Asn60, Asp49, Asp43, and Gln58, all interacting with the hydroxy groups of the rings of the compounds. Additionally, the unique compound interacts with Trp109 between the rings of the compounds. It is interesting because it is the only compound that promotes the formation of amyloid fibers, as demonstrated by SEM microscopy. This point suggests that the interactions between rings could be the reason for the proliferation of amyloid fibers.

## 4 Conclusion

The current research is a significant step in understanding the effects of eight different phenolic compounds on the fibrillogenesis of human lysozyme. Among these, only two compounds, caffeic acid and l-tyrosine, have been found to inhibit the formation of amyloid fibers. Both compounds reduced fluorescence in ANS and ThT assays, but ANS and CD analyses show that the structures produced differ. The remaining six compounds do not inhibit the fibrillogenesis of lysozyme and either increase or maintain fibrillogenesis at similar levels. The structures generated by these compounds also vary. Docking experiments indicate that the interactions between the phenolic compounds and specific residues of lysozyme—Glu35 and Trp64—are crucial for an inhibitory effect. The chemical structure of phenolic compounds should include a phenolic ring, a short R chain, and a molecular weight of around 180. Overall, this study demonstrates that all phenolic compounds tested influence the fibrillogenesis of human lysozyme. This finding encourages further exploration of various phenolic compounds in the search for potential drugs to treat amyloidosis-related diseases.
